# Knowledge, attitudes and practices on diabetic foot care among nurses in Kimberley, South Africa

**DOI:** 10.4102/safp.v66i1.5935

**Published:** 2024-06-25

**Authors:** Labala G. Mafusi, Chika K. Egenasi, Wilhelm J. Steinberg, Mathew O. Benedict, Talat Habib, Melvin Harmse, Cornel van Rooyen

**Affiliations:** 1 Department of Family Medicine, Faculty of Health Sciences, University of the Free State, Bloemfontein, South Africa; 2 Department of Family Medicine, Robert Mangaliso Sobukwe Hospital, Kimberley, South Africa; 3 Department of Biostatistics, Faculty of Health Science, University of the Free State, Bloemfontein, South Africa

**Keywords:** diabetic foot, nurses, Sol Plaatje, knowledge, attitudes, practices, guidelines, clinics

## Abstract

**Background:**

Diabetic foot is a dangerous complication of diabetes and can lead to high morbidity and mortality. As essential team members of the healthcare system, nurses play an important role in diabetic foot management and are indispensable in patients’ education to prevent diabetic foot. The study assessed nurses’ knowledge, attitudes and practices regarding diabetic foot care in Sol Plaatje primary health care centres in the Northern Cape: Sol Plaatje’s 14 district municipality clinics, Kimberley, Northern Cape.

**Methods:**

This was a descriptive cross-sectional analytical study. A questionnaire assessed nurses’ knowledge, practices and attitudes towards diabetic foot care in the above setting.

**Results:**

A total of 128 professionals, enrolled and auxiliary nurses who are providing primary care to patients within the 14 clinics in the Sol-Plaatje sub-district were recruited for the study. Hundred and five participants completed the self-administered questionnaires. The majority (95%) were females and 58.1% knew that South African Diabetic Foot Guidelines existed, while 57.7% had read them. About 57% did not know about the 60-s diabetic foot screening tool, and 67% did not know the 10 g monofilament test. Approximately 29.8% had never attended a class on diabetic foot care and 85.6% required training on diabetic foot care.

**Conclusion:**

This study revealed that the majority of nurses working in the Sol-Plaatje sub-district primary health care centres are knowledgeable of the diabetic foot guidelines for primary care. However, there is a need for ongoing education on diabetic foot care.

**Contribution:**

The study results will help improve nurses’ awareness of the importance of diabetic foot care.

## Introduction

Diabetes-related foot disease (DFD) is the leading cause of amputation, hospitalisation, poor quality of life and disability burden worldwide.^1^ According to the 2023 International Working Group on the Diabetic Foot (IWGDF) Guidelines, the condition is defined as:

[*D*]isease of the foot of a person with current or previously diagnosed diabetes mellitus that includes one or more of the following: peripheral neuropathy, peripheral artery disease, infection, ulcer(s), neuro-osteoarthropathy, gangrene, or amputation.^2^

Based on current estimates, DFD contributes around 2% of the global disease burden, making it the 13th leading cause of disease burden from more than 350 conditions.^1^ Foot ulcers associated with diabetes are among the most serious diabetic complications, placing a substantial burden on the person’s family, healthcare professionals and society at large.^3^ Several factors increase the risk of complications, such as poor glycaemic control, smoking, foot deformities, peripheral neuropathy, visual loss and chronic kidney disease.^4,5^ With effective screening, risk assessment and meticulous foot care, these complications can be proactively delayed or prevented.^5^ This highlights the significance of early intervention and comprehensive foot care practices in mitigating the progression of DFD among diabetic individuals.

A systemic review and meta-analysis on characteristics, prevalence and outcomes of diabetes-related foot ulcers (DFU) in Africa included 56 173 diabetic patients from 19 African countries. They found a foot ulcer prevalence of 13%.^6^ Approximately 15% of these patients needed major amputations, and 14.2% died during in-hospital admissions. In sub-Saharan Africa, the DFU prevalence is estimated to range from 7.2% to 13.0%.^6,7^ However, the estimated prevalence in Ethiopia varies from 12% to 32%.^8,9,10,11^ Screening and educational programmes and interventions that evaluate foot vascular and neurological status play important roles in reducing or preventing diabetic foot disease progression in the African continent.^6^

A descriptive study in KwaZulu-Natal by Somasundaram et al. found that the incidence of lower limb amputation was rising, especially in urban areas.^12^ Diabetes mellitus was a major reason for atraumatic foot amputation. In South Africa, patients lack access to a multidisciplinary foot care team and have less access to screening for foot problems. The authors suggested that emphasis should focus on prevention and strategy to delay lower limb amputation through improved diabetes control, increased foot education and the introduction of specialised foot clinics.^12^

The primary prevention objective should aim at protecting the healthy population from getting a disease or sustaining an injury. Prevention, identification and treatment of diabetes-related complications are achieved better with early recognition of the disease and education.^13,14,15^ Regular monitoring for complications consists of a series of tests and skin-focused evaluations aimed at detecting complications at an early stage of diabetes.^3,16^

Effective prevention of complications of DFD necessitates active patient involvement and comprehensive education about their condition, enabling early identification and appropriate response to DFD.^17^ The importance of daily self-examination of the feet cannot be overstated, emphasising the need for vigilant monitoring of any abnormalities.^18^ Healthcare practitioners must actively advocate smoking cessation, particularly among patients with chronic wounds and arterial insufficiency, during every encounter.^19^

The clinician’s commitment to diabetic foot prevention significantly influences the quality of care provided. Variables such as knowledge and attitude are pivotal in determining the extent of involvement in diabetic foot care. Comprehensive training sessions and programmes are essential to enhance health care providers’ proficiency and engage nurses actively in both theoretical and practical aspects of diabetic foot care and management. Additional educational resources, including virtual diabetes clinic platforms, continuing professional development, printed educational materials, educational outreach, didactic formats, audit feedback and adherence to diabetic foot guidelines, along with multifaceted interventions, prove to be effective methodologies for educating healthcare professionals.^20,21,22,23,24^

A previous study evaluated the knowledge, attitudes and practices (KAPs) of adult patients with type 2 diabetes at primary health care (PHC) clinics in Kimberley, Northern Cape. The study demonstrated good diabetes-related knowledge and attitudes but inadequate practices among participants.^25^ While there was a guideline published by the Society for Endocrinology, Metabolism and Diabetes of South Africa (SEMDSA) to guide diabetic foot care in South Africa,^16^ no study had been done to assess knowledge of the guidelines by public healthcare workers in the Northern Cape province. The current study aimed to determine nurses’ KAPs on diabetic foot care and prevention in Sol-Plaatje sub-district public health facilities in Northern Cape, South Africa.

## Research methods and design

### Study design

This was a descriptive cross-sectional analytical study using a self-administered questionnaire.

### Study setting

The study was conducted in public health centres within Sol Plaatje Municipality in the Frances Baard District, Northern Cape. Sol Plaatje sub-district comprises 14 clinics, namely: Galeshewe Community Health Centre, Ritchie, City Clinic, Beaconsfield, Floors Clinic, Betty Gaetsewe, Masakhane, Mapule Matsepane, Dr. Winston Torres, Platfontein, Madoyle Clinic, Phutanang, Greenpoint and Riverton Clinic. Northern Cape is the least populated province in South Africa, with an estimated population of 1 263 875 as of 2019. Approximately, 31% of the populace is younger than 15 years, 64% is between 15 years and 64 years and 5% is over 60 years. The province has the largest land area in the country, with a surface area of 372 889 km^2^.^26^

### Study population

The study population consists of 128 individuals taken from the human resource database from the 14 clinics in the Sol Plaatje sub-district. The study included all nurses working in the 14 clinics. The targeted population comprises 104 professional nurses, 5 enrolled nurses and 19 auxiliary nurses.

### Inclusion and exclusion criteria

Professional, enrolled or auxiliary nurses working in one of the 14 public clinics in Sol Plaatje District Municipality who consented were included in the study.

### Sample size

The intended sample for the study consisted of a maximum of 128 participants. Feedback was received from 105 participants – 92 professional nurses, 3 enrolled nurses, 9 auxiliary nurses and 1 unspecified participant, with a response rate of 82%. The remaining 18 of the sample could not be reached either because they were on annual leave or quarantine or isolation from coronavirus disease 2019 (COVID-19), thus absent during visits to their respective clinics. One was discarded for incorrectly filling the forms, and four declined to give consent. The study included everyone who satisfied the inclusion criteria; a power calculation to determine an adequate sample size was unnecessary.

### Data collection

Self-administered questionnaires were used to gather data from the participants. The questionnaire was designed based on information from the literature on diabetic foot care.^16,27,28^ Further validation was done internally by the Department of Family Medicine at the University of the Free State.

The questionnaire consisted of four sub-sections designed to assess the following:

- Nursing staff’s demographic variables (gender, age, professional qualification and years of experience), knowledge of diabetic foot care, attitudes towards the practice of informing patients with diabetes about foot care and examining their feet.
- The ‘knowledge’ section comprised 30 questions designed to evaluate the nurse’s level of knowledge on diabetic foot complications, diabetic foot-associated risks, diabetic foot examination and diabetic foot prevention. The questions were statements that required respondents to state ‘true’ ‘false’ or ‘don’t know’. Knowledge was categorised using Bloom’s cut-off point. This categorisation grades 80% and above as good knowledge, 60% – 79% as moderate and less than 60% as poor knowledge.^29^
- The ‘attitude’ section consisted of 11 items with options strongly agree, agree, neutral, disagree and strongly disagree, designed to assess the following: constraints to provide diabetic foot education during routine consultations and attitude of nurses on diabetic foot examination in foot care.
- The ‘practice’ section consists of 12 items requiring a Yes or No response. The items assess the training routines of nurses concerning diabetic foot care and teaching patients with diabetes about lifestyle modifications.
- The first author and his assistant distributed the questionnaires to clinic participants after explaining the research’s nature and purpose.

### Pilot study

The pilot study was done at City Clinic in the last week of April 2020. Four professional nurses and one auxiliary nurse completed the questionnaires. No amendment was necessary, and data from the pilot study were not included.

### Statistical analysis

The collected data were computed on an Microsoft Excel spreadsheet and analysed using SAS 9.4 software. Descriptive statistics were calculated, namely cumulative frequencies and percentages for categorical data and median. The results are presented in tables and graphs, and the association between variables was determined using appropriate statistical interpretations. Chi-square was used to determine the association between participants’ level of education and KAPs. The probability value (*p*-value) of less than 0.05 was accepted as statistically significant.

### Ethical considerations

Ethical approval to conduct the study was obtained from the Health Sciences Research Ethics Committee (HSREC) of the University of the Free State with reference number UFS-HSD2019/1884/2104. Approval for data collection was also obtained from the Northern Cape Health Department identification number NC_2020SOL_001.

Number coding was used to ensure the confidentiality of the participants’ responses. No names or personal identifiers appeared on any research-related information or datasheet sent for statistical analysis. All paper-based records were kept in a secure location by the researcher and were only accessible to those involved in the study. All information was managed in a strictly professional and confidential manner.

## Results

A total of 105 participants were recruited for the study with a response rate of 82%, and one participant had some missing data. The majority were females (95.2%) with a minimum age of 24 years, a maximum age of 73 years and a median age of 48 years.

Table 1 indicates that more participants were females in the age group 51–60 years and were professional nurses with over 10 years of experience.

**TABLE 1:** Demographic characteristics of participants.

Variables	*n*	%
**Gender**		
Male	5	4.8
Female	99	95.2
**Age category**		
20–30	9	8.7
31–40	22	21.2
41–50	31	29.8
51–60	36	34.6
> 60	6	5.8
**Profession/rank**		
Professional nurse	92	88.5
Enrolled nurse	3	2.9
Auxiliary nurse	9	8.7
**Year of practice**		
0–5	19	18.8
6–10	14	13.9
> 10	68	67.3

Table 2 shows the participants’ knowledge scores. The majority know of the diabetic foot guideline for PHC professionals but are unaware of the 60-s screening tool for high-risk diabetic foot.

**TABLE 2:** Participant’s knowledge score.

Knowledge item		True	%	False	%	Do not know	%
1.	South Africa has a diabetic foot guideline for primary healthcare professionals	61	58.1	8	7.6	36	34.30
2.	Diabetic foot is associated with neurological and vascular damages	95	90.0	6	6.0	4.0	4.00
3.	A 60-s test is a screening tool for low-risk diabetic foot	45	43.0	14	13.0	46	44.00
4.	A history of the previous ulcer is a high-risk diabetic foot only if both feet were affected	36	34.3	57	54.3	12	11.40
5.	Previous amputation of only one toe is not a high-risk diabetic foot	11	10.5	82	78.1	12	11.40
6.	The absence of foot pulses is a high risk only if both dorsalis pedis and posterior tibialis pulses are affected	47	44.7	49	46.7	9	8.60
7.	Calluses, blisters, fissures and ulcers are high-risk diabetic foot but not the ingrowing toenail	33	31.4	68	64.8	4	3.80
8.	Smoking cessation is not important to prevent diabetic foot	16	15.2	87	82.9	2	1.90
9.	High blood sugar and cholesterol are high risk diabetic foot	97	92.4	6	5.7	2	1.90
10.	Weight loss can reduce the incidence of diabetic foot	84	80.0	16	15.2	5	4.90
11.	Poor blood supply to the legs can increase diabetic foot risk	105	100.0	0	0.0	0	0.00
12.	Charcot foot is a foot deformity caused by significant nerve damage	64	61.0	4	4.0	37	35.00
13.	Foot deformity alone is not enough risk for specialist referral	22	20.9	76	72.4	7	6.70
14.	Specialist referral can be delayed if there is only foot deformity but pulses are present	48	45.7	49	46.7	8	7.60
15.	Specialist referral can be delayed if there is only an active ulcer, but pulses are present	35	33.3	64	61.0	6	5.70
16.	Absence of pulse needs urgent referral but not an active ulcer or bony deformity	50	47.6	47	44.8	8	7.60
17.	A negative result for the ‘60-second Foot Screening Tool’ does not require referral to the specialist	36	34.3	39	37.1	30	28.60
18.	Part of the foot exam consists of checking 4th and 5th web spaces and nails	71	67.6	22	21.0	12	11.40
19.	10g monofilament is used to check for nerve damage in the foot	35	33.0	6	6.0	64	61.00
20.	Diabetic patients should be encouraged to sit with their legs crossed	7	6.7	87	82.9	11	10.50
21.	Diabetic patients should not dry between their toes unless they have an obvious ulcer	10	9.5	93	88.6	2	1.90
22.	Diabetic patients should trim their toenails only if it is painful	8	7.6	93	88.6	4	3.80
23.	Diabetic patients should not inspect their shoes prior to wearing them if their sugar is well controlled	8	7.6	96	91.4	1	1.00
24.	Diabetic patients should not bother to wash their feet everyday if it is not dirty	3	2.9	99	94.2	3	2.90
25.	Diabetic patients should always check the water temperature before washing feet	98	93.3	4	3.8	3	2.90
26.	Diabetic patients are allowed to walk barefoot only on clean surfaces	15	14.2	85	81.0	5	4.80
27.	Diabetic foot patients already referred and waiting for a specialist appointment do not need any education on what changes to observe while awaiting	4	3.8	99	94.3	2	1.90
28.	Diabetic patients need education only if they have foot problems	7	6.7	95	90.5	3	2.90
29.	Diabetic patients should not wear tight shoes but very ample non-fitting shoes	61	58.1	40	38.1	4 (3.8)	4 (3.8)
30.	A patient with negative results for the ‘60-Second Foot Screening Tool’ needs to be examined yearly	45	42.8	28	26.7	32 (30.5)	32 (30.5)

Of the majority of respondents (*n* = 70), 66.7% had moderate knowledge (60% – 79%), 18.1% (*n* = 19) had good knowledge (80% – 100%), while 15.2% (*n* = 16) had poor knowledge (< 60%).

Table 3 shows the participants’ attitude scores, with almost every participant accepting that diabetic education is an important aspect of their job.

**TABLE 3:** Participants’ attitude score.

Item	Strongly agree (%)	Agree (%)	Neutral (%)	Disagree (%)	Strongly disagree (%)
I think diabetes control is more important than preventing foot problem	42.3	16.4	10.6	17.3	13.5
Diabetic education is an important part of my job	80.9	17.1	1.0	0.0	1.0
It is not worth educating a patient who has already developed an ulcer	1.0	1.0	3.8	32.7	61.5
I do not think patients with diabetic foot problems are my concern	2.9	4.7	1.0	19.0	72.4
Diabetic education is a waste of time as patients are not receptive to healthcare providers’ education	1.0	1.9	1.9	27.6	67.6
I do not educate diabetic patients on foot problem because it is time consuming	1.0	3.8	0.0	20.0	75.2
I do not have sufficient time to advise each patient individually on how to look after their feet	3.8	18.3	7.7	29.8	40.4
It is not necessary to assess diabetic foot regularly	1.9	2.9	1.0	28.5	65.7
Diabetic patients should have their foot examination recorded in their files at each visit to the primary healthcare facility	49.5	35.2	4.8	3.8	6.7
I do not like to examine patient’s feet as it stinks	0.0	3.9	4.9	29.4	61.8
I think foot care awareness is important in self-care	72.8	18.5	0.0	2.9	5.8

Table 4 shows the perceived practice score for participants; more than half read the diabetes management guidelines, but less than half recorded the foot examination findings in their patient files.

**TABLE 4:** Participants’ practice score.

Practice item		Yes (%)	No (%)
1.	Have you ever read the diabetes management guidelines for primary healthcare providers?	57.7	42.3
2.	Have you ever attended a class on how to care for a diabetic patient’s foot problem?	29.8	70.2
3.	I do record in the file the foot examination of diabetic patients attending my facility	46.1	53.9
4.	I do ask patients about their foot problems at each visit	62.1	37.9
5.	I do practice a 60-s screening tool assessment for all my diabetic patients	17.6	82.4
6.	I do check patient’s feet for loss of sensation	64.4	35.6
7.	I do check diabetic patient’s feet for any deformities, calluses, infections or ulcers at each visit	57.7	42.3
8.	I do check diabetic patient’s footwear at each visit	54.8	45.2
9.	I always discuss diet with my diabetic patients	89.4	10.6
10.	I educate and encourage diabetic patients on smoking cessation	90.4	9.6
11.	Do you think you need training in diabetic foot care?	85.6	14.4
12.	I do not have wound care experience	40.4	59.6

Table 5 shows the association between the background characteristics and KAP. Knowledge was not significantly associated with participants’ profession or years of practice.

**TABLE 5:** Bivariate analysis of background characteristics versus knowledge, attitudes and practices.

KAP items	Scale	Demographic characteristics							
		Profession/rank			*p*	Years of practice			*p*
		Professional nurse (%)	Enrolled nurse (%)	Auxiliary nurse (%)		0–5 (%)	6–10 (%)	> 10 (%)	
Knowledge	Poor	11.9	33.3	44.40	0.062	36.8	21.4	8.8	0.051
	Moderate	67.4	66.7	55.60	-	47.4	57.1	72.1	-
	Good	20.7	0.00	0.00	-	15.8	21.4	19.1	-
**Attitude**									
Diabetic education is an important part of my job	Strongly agree	91.7	2.3	6.00	0.191	19.5	11.0	69.5	0.068
	Agree	72.2	5.6	22.20	-	11.8	23.5	64.7	-
	Neutral	100.0	0.0	0.00	-	100	0.0	0.0	-
	Disagree	-	-	-	-	-	-	-	-
	Strongly disagree	100.0	0.0	0.00	-	0.0	100	0.0	-
I do not think patients with diabetic foot problems are my concern	Strongly agree	100.0	0.0	0.00	**0.004**	100	0.0	0.0	0.109
	Agree	100.0	0.0	0.00	-	0.0	0.0	100	-
	Neutral	75.0	25.0	0.00	-	50.0	25.0	25.0	-
	Disagree	73.5	2.9	23.50	-	8.8	17.7	73.5	-
	Strongly disagree	96.8	1.6	1.60	-	21.7	11.7	66.7	-
It is not worth educating a patient who has already developed an ulcer	Strongly agree	100.0	0.0	0.00	0.209	0.0	0.0	100	0.760
	Agree	75.0	0.0	25.00	-	25.0	25.0	50.0	-
	Neutral	100.0	0.0	0.00	-	0.0	0.0	100	-
	Disagree	75.0	10.0	15.00	-	10.0	20.0	70.0	-
	Strongly disagree	92.1	1.3	6.60	-	21.9	12.3	65.8	-
I do not educate diabetic patients on foot problems because it is time consuming	Strongly agree	100.0	0.0	0.00	**0.034**	0.0	0.0	100	0.856
	Agree	100.0	0.0	0.00	-	25.0	25.0	50.0	-
	Neutral	-	-	-	-	-	-	-	-
	Disagree	66.7	9.5	23.80	-	15.0	15.0	70.0	-
	Strongly disagree	93.6	1.3	5.10	-	19.7	13.2	67.1	-
Diabetic patients should have their foot examination recorded in their files at each visit to the primary healthcare facility	Strongly agree	96.1	2.0	1.90	**0.043**	14.3	8.2	77.5	0.090
	Agree	83.8	5.4	10.80	-	24.3	16.2	59.5	-
	Neutral	80.0	0.0	20.00	-	20.0	40.0	40.0	-
	Disagree	50.0	0.0	50.00	-	25.0	50.0	25.0	-
	Strongly disagree	85.7	0.0	14.30	-	16.7	0.0	83.3	-
**Practice**									
I do record in the file the foot examination of diabetic patients attending my facility	Yes	89.4	4.3	6.40	0.543	13.0	10.9	76.1	0.281
	No	87.5	1.8	10.70	-	24.1	14.8	61.1	-
I do practice a 60-s screening tool assessment for all my diabetic patients	Yes	83.3	0.0	16.70	0.220	11.1	16.7	72.2	0.516
	No	90.4	3.6	6.00	-	21.3	11.2	67.5	-
I do check diabetic patient’s footwear at each visit	Yes	87.5	1.8	10.70	0.543	14.3	19.6	66.1	0.050
	No	89.4	4.3	6.30	-	25.0	4.6	70.4	-
I do not have wound care experience	Yes	90.2	4.9	4.90	0.366	7.5	17.5	75.0	**0.039**
	No	87.1	1.6	11.30	-	26.7	10.0	63.3	-

Note: Bold values, statistically significant difference.

KAP, knowledge, attitudes and practices.

There was no significant association between knowledge and category of nursing staff (*p* = 0.062) nor knowledge and years of practice of the participants (*p* = 0.051). There was a significant association between the attitude item ‘I don’t think patients with diabetic foot problem are my concern’ and the profession of the participants (*p* = 0.004) but not with the years of practice of the participants (*p* = 0.109). Attitude items ‘I do not educate diabetic patients on foot problems because it is time consuming’ and ‘Diabetic patients should have their foot examination recorded in their files at each visit to the primary healthcare facility’ were significantly associated with the profession of participants (*p* = 0.034) and (*p* = 0.043), respectively.

The practice item ‘I do not have wound care experience’ was significantly associated with the participant’s years of practice (*p* = 0.039). Participants with more years of practice were more likely to have better wound care experience than those with fewer years of practice.

Table 6 evaluates the association between KAP items. It indicates that knowledge was significantly associated with the item ‘It is not worth educating a patient who has already developed an ulcer’.

**TABLE 6:** Associations between knowledge, attitudes and practices.

Items	Scale	Knowledge			*p*
		Poor (%)	Moderate (%)	Good (%)	
**Attitude**					
Diabetic education is an important part of my job	Strongly agree	11.8	69.4	18.8	**0.049**
	Agree	27.8	61.1	11.1	-
	Neutral	0.0	0.0	100	-
	Disagree	-	-	-	-
	Strongly disagree	100.0	0.0	0.0	-
It is not worth educating a patient who has already developed an ulcer	Strongly agree	100.0	0.0	0.0	**0.002**
	Agree	100.0	0.0	0.0	-
	Neutral	75.0	0.0	25.0	-
	Disagree	14.7	73.5	11.8	-
	Strongly disagree	9.4	70.3	20.3	-
I do not think patients with diabetic foot problems are my concern	Strongly agree	0.0	33.3	66.7	0.124
	Agree	40.0	40.0	20.0	-
	Neutral	0.0	0.0	100	-
	Disagree	15.0	65.0	20.0	-
	Strongly disagree	14.5	71.0	14.5	-
I do not educate diabetic patients on foot problems because it is time consuming	Strongly agree	0.0	100	0.0	0.612
	Agree	50.0	50.0	0.0	-
	Neutral	-	-	-	-
	Disagree	14.3	71.4	14.3	-
	Strongly disagree	13.9	65.8	20.3	-
Diabetic patients should have their foot examination recorded in their files at each visit to the primary healthcare facility	Strongly agree	7.7	69.2	23.1	0.115
	Agree	18.9	70.3	10.8	-
	Neutral	20.0	40.0	40.0	-
	Disagree	50.0	50.0	0.0	-
	Strongly disagree	28.6	57.1	14.3	-
**Practice**					
I do record in the file the foot examination of diabetic patients attending my facility	Yes	14.6	70.8	14.6	0.747
	No	16.1	64.3	19.6	-
I do practice a 60-s screening tool assessment for all my diabetic patients	Yes	16.7	55.6	27.7	0.398
	No	15.5	69.0	15.5	-
I do check diabetic patient’s footwear at each visit	Yes	15.8	61.4	22.8	0.238
	No	14.9	74.5	10.6	-
I do not have wound care experience	Yes	11.9	69.1	19.0	0.701
	No	17.7	66.1	16.2	-

Note: Bold values, statistically significant difference.

A significant association was found between the following attitude items and knowledge: ‘Diabetic education is an important part of my job’ and ‘It is not worth educating a patient who has already developed an ulcer’, with *p*-values of 0.049 and 0.002, respectively.

## Discussion and recommendations

Adequate knowledge of diabetic foot care is crucial in managing patients with diabetes. Guidelines such as the SEMDSA guidelines for diabetic foot care for primary care professionals can assist healthcare workers with the required information to manage patients better.^18^

This study assessed the KAPs of nursing staff at the Northern Cape, Sol Plaatje sub-districts primary health clinics regarding diabetic foot care.

### Knowledge

A considerable number of the participants are unaware of the existence of the South African Diabetic Foot guideline, which is available online and may explain the sub-optimal level of knowledge among some of the participants in this study. An Australian study by Schoen et al. also found that the availability of diabetes foot care education brochures was low, particularly in rural settings.^30^ There is, therefore, the need to increase nursing staff awareness of the guideline’s availability, with an emphasis on the importance of disseminating this information for improved diabetic foot care in the study setting.

The 60-s screening tool was developed by Sibbald et al. to identify high-risk diabetic foot status.^27^ The tool is designed to quickly assess diabetic foot features, and a positive response to any item indicates the need for referral for specialist assessment.^31^ In the study setting, because of the unavailability of vascular surgeons, patients with high-risk diabetic foot features would be referred to general surgeons for management.

The study participants’ awareness of this screening tool is questioned, with indications that knowledge about its purpose and application may be insufficient. Only very few knew that the test was not to identify low-risk but high-risk diabetic feet. Poor knowledge was also shown in the response to some other items relating to this screening tool. For example, over half of the participants view a patient as high risk for diabetic foot only if both dorsalis pedis and posterior tibialis arteries pulses are absent. Periodic continuing healthcare provider education and training on this subject by healthcare professionals knowledgeable about diabetic foot care in collaboration with organisations such as SEMDSA may be needed. An educational intervention among primary care nurses in Brazil was shown to improve knowledge of diabetic foot care.^32^

It was, however, applaudable that the majority of the participants were aware that diabetic foot is associated with neurological and vascular complications. The majority also knew that smoking cessation, weight reduction and good blood supply can decrease the risk of diabetic foot. The participants’ knowledge of diabetic foot care and relevant safety measures was also satisfactory. Regarding foot examination, most participants knew the need to inspect the 4th and 5th web spaces. There was, however, a poor knowledge of the 10 g monofilament used to check for neuropathy.

A study by Albagawi et al.^33^ showed that nurses can play a crucial role in enhancing patients’ understanding, particularly in the context of diabetic care. Implementing educational programmes can significantly enhance the quality of life for individuals with diabetes by fostering self-care practices, particularly in relation to foot care.^34^ Furthermore, nurses are strategically positioned to identify high-risk patients within the community, often overseeing community health workers, thus contributing to preventing or delaying diabetes-related foot issues.^20^ The suboptimal knowledge regarding the screening and examination of diabetic foot among some PHC nurses in the study setting raises significant concerns. Given that most PHC centres serve as the initial point of contact for patients and are predominantly nurse-driven,^35,36^ the lack of adequate knowledge is particularly problematic. The failure or delay in referring high-risk patients could have serious consequences for their health and wellbeing. Urgent attention and targeted interventions, such as online diabetic education and training classes at a regular frequency and leaflet distribution to healthcare workers and patients, are necessary to address these knowledge gaps and enhance the capabilities of PHC nurses in managing diabetic foot issues effectively.

Overall, it was reassuring that most (84.8%) of the study participants had moderate to good knowledge about diabetic foot care. There is, therefore, a foundation to build upon and potential for improvement through targeted interventions and education.

### Attitudes

In this study, most participants strongly agree that educating diabetic patients is an important part of their jobs. A systemic review by Nikitara et al. supports the role of nurses as educators and the positive outcome of diabetic education on patients’ glycemic control when nurses play this role.^37^ Chawla et al., in a case-control study in India, randomised patients to a case and control group; the case group received education on diabetes while the control group did not. They were followed up, and there was a significant decline in random blood glucose in the case group on follow-up.^38^ This shows that effective health education can help maintain better glucose control and slow down disease progression and complications. Most nurses are trained to educate patients about various disease conditions, which would have influenced their positive responses. Most of the participants disagreed with the statement that it is not worth educating patients who already have foot ulcers. This was supported by Raju et al., who reported in their study about educational interventions and diabetic foot ulcers that educational interventions have reduced the severe foot complications and incidence of amputations associated with diabetic foot ulcers.^39^ Most participants also disagree that diabetic education of patients is a waste of time; this was affirmed in a study by Manickum, Madiba and Ramklass, which showed that diabetic foot education effectively changed behaviours in patients with diabetes mellitus.^40^ Lack of time on the part of the healthcare practitioners was not reported as a barrier to educating patients about foot care in the various studies reviewed.^41,42^ This is in keeping with our study, where most of our participants disagreed with the statement that they do not educate patients with diabetes on diabetic foot problems because it is time-consuming. However, in some studies, authors and patients reported a lack of time on the side of healthcare practitioners as a barrier to diabetic foot care.^43,44^

Most participants disagree with the statement, ‘I don’t think patients with diabetic foot problems are my concern’. Song and Chambers reported that intensive diabetic foot care and education by nurses reduce the amputation rate for high-risk diabetic feet and help control blood glucose.^45^ In our study, some participants agree with this statement because, in certain instances, these patients are sent to specialists or podiatrists for management.^18^ In their study, Hill, Ellis and Gillison described different views of healthcare providers on the division of foot care responsibilities. Some practitioners prefer to pass on the responsibilities to the podiatrist as they are more focused on foot care.^42^

Gallman, Corner and Johnson reported in a study about improving the detection of foot abnormalities in patients with diabetes that routine foot examinations should be completed and correctly documented using electronic medical records in the PHC setting.^44^ This is in keeping with the response of our participants, who agree that patients with diabetes should have their foot examination recorded in their files at each visit to the PHC facility.

Most participants disagreed with the statement, ‘I do not like to examine patients’ feet as it stink’, which contrasts with other studies on nurses’ feelings about malodorous wounds; Ousey and Roberts reported that when healthcare workers come close to exudating malodorous wounds, it becomes stressful to remain close to these patients. They strive to protect the patient’s vulnerability and the healthcare workers’ defenselessness’. In our study, the nurses may have found it embarrassing to admit that bad odour affects their examination of diabetic feet because healthcare workers are expected to learn how to cope with bad odour.^46^

Almost all participants agree that foot care awareness is important for patient self-care; this was supported by most of the studies reviewed.^42,43,47^

### Practices

More than half of the participants admitted that they had read the diabetes management guidelines for primary care providers. In a study on diabetes care and management by Albagawi et al., they reported that nurses with access to diabetes management guidelines performed better than their peers.^33^ About two-thirds of our participants had never attended a class on how to care for patients with diabetic foot problems, and most responded ‘Yes’ to the question ‘Do you need training in diabetic foot care?’ despite a majority indicating that they have wound care experience. These are similar to findings in a study by Kaya and Karaca on the evaluation of nurses on diabetic foot care; it was reported that only one-third (34%) of the nurses enrolled in the study were trained in diabetic foot care, 77.5% did not perform diabetic foot examination on patients and 42.8% stated they need training in diabetic foot care.^20^ The reason for this in our study may be the lack of specific training programmes on the knowledge and practical applications of diabetic foot care.

A majority of our participants, even though they agree that patients should have their foot examination recorded in their files, more than half of them said that they do not record the foot examination in their patients’ files. Documenting the findings of patients’ foot examinations in the patient’s records is important.^44^ If it is not being documented, it was probably not done.

The majority of our participants say that they do ask and also check patients’ feet for loss of sensations, deformities, calluses, ulcers and footwear. This practice is also stipulated in the SEMDSA diabetic foot care guidelines for primary health care professionals,^18^ which healthcare practitioners must follow. Hidalgo-Ruiz et al., in a study on the assessment of diabetic foot prevention by nurses in Spain, reported that 96.58% asked their patients to remove their footwear, 78.34% performed thorough examinations such as footwear, socks, temperature, pain, changes in skin colour on the feet, presence of oedema and foot deformities, and 80.25% assessed the risk of developing diabetic foot. Barefoot examination was performed at each visit by 36.31% of participants.^48^ This contrasted with findings by Kaya and Karaca on the evaluation of nurses on diabetic foot care, where 77.5% of nurses do not perform foot examinations for patients in their units.^20^

Most of the participants in the study did not practice using the 60-s screening tool for their diabetic patients; this is a well-known tool that has been piloted and validated.^27^ It has been modified and is used by nurses and other healthcare professionals in many countries, such as Canada and Guyana.^49^ The participants in our study probably did not know about the existence of the screening tool because of poor education on diabetic foot care.

Most of the study participants say that they continually educate patients on diets and smoking cessation. Song and Chambers discussed the positive role of intensive patient education by nurses; educating patients with diabetic feet about diet and other lifestyle modifications helps reduce the risk of foot ulceration and amputation.^45^ This supports the findings of our study: nurses are usually trained to provide self-management healthcare advice to patients about various medical conditions. In contrast, Kaya and Karaca, in their study on the evaluation of nurses on diabetic foot care, reported that only 18.6% of nurses in the study teach patients about blood sugar control.^20^

## Conclusion

This study found that most participants from Sol Plaatje Municipality within the Northern Cape need to acquire knowledge of existing diabetic foot guidelines and the 60-s foot screening tool. However, most nurses within this municipality know about diabetic foot complications and associated risk factors.

These findings suggest that PHC workers need increased awareness, training and education about diabetic foot care guidelines and the 60-s screening test to improve patient care and prevent foot complications.

### Strengths and limitations of the study

The study benefited from a high response rate from the required number of participants, which enhanced the accuracy and data quality. However, because of the COVID-19 pandemic, some participants could not be reached. Also, the study could only focus on nursing staff as doctors only do outreach in 3 of the 13 clinics. As the study was conducted in only one municipality in the Northern Cape, the external validity of applying these findings to the entire Northern Cape and South Africa is limited. Furthermore, using the self-reported methods and non-homogenous groups of participants may have introduced bias, as they might have over-reported their attitudes and practices in the provision of diabetic foot care. Additionally, the study’s ability to differentiate between the expected knowledge levels for each professional group was limited, as a professional nurse may be more likely to possess better diabetic foot care guideline knowledge than enrolled or auxiliary nurses.
